# Impact of *Babesia microti* infection on the initiation and course of pregnancy in BALB/c mice

**DOI:** 10.1186/s13071-021-04638-0

**Published:** 2021-03-02

**Authors:** Katarzyna Tołkacz, Anna Rodo, Agnieszka Wdowiarska, Anna Bajer, Małgorzata Bednarska

**Affiliations:** 1 Department of Eco-epidemiology of Parasitic Diseases, Institute of Developmental Biology and Biomedical Sciences, Faculty of Biology, University of Warsaw, 1 Miecznikowa Str, 02-096 Warsaw, Poland; 2 Institute of Biochemistry and Biophysics, Polish Academy of Sciences, 5A Pawińskiego Str, 02-106 Warsaw, Poland; 3 Department of Pathology and Veterinary Diagnostics, Faculty of Veterinary Medicine, Warsaw University of Life Sciences-SGGW, 159 Nowoursynowska Str, 02-776 Warsaw, Poland; 4 PulsVet Specialist Veterinary Clinic, 7 Alternatywy Str, 02-775 Warsaw, Poland; 5 Department of Parasitology, Institute of Functional Biology and Ecology, Faculty of Biology, University of Warsaw, 1 Miecznikowa Str, 02-096 Warsaw, Poland

**Keywords:** *Babesia microti*, Congenital babesiosis, Emerging tick-borne disease, Experimental model, Poland, Pregnancy, Vertical transmission

## Abstract

**Background:**

Protozoa in the genus *Babesia* are transmitted to humans through tick bites and cause babesiosis, a malaria-like illness. Vertical transmission of *Babesia* spp. has been reported in mammals; however, the exact timing and mechanisms involved are not currently known. The aims of this study were to evaluate the success of vertical transmission of *B. microti* in female mice infected before pregnancy (mated during the acute or chronic phases of *Babesia* infection) and that of pregnant mice infected during early and advanced pregnancy; to evaluate the possible influence of pregnancy on the course of parasite infections (parasitaemia); and to assess pathological changes induced by parasitic infection.

**Methods:**

The first set of experiments involved two groups of female mice infected with *B. microti* before mating, and inseminated on the 7th day and after the 40th day post infection. A second set of experiments involved female mice infected with *B. microti* during pregnancy, on the 4th and 12th days of pregnancy. Blood smears and PCR targeting the 559 bp 18S rRNA gene fragment were used for the detection of *B. microti*. Pathology was assessed histologically.

**Results:**

Successful development of pregnancy was recorded only in females mated during the chronic phase of infection. The success of vertical transmission of *B. microti* in this group was 63%. No evidence of pregnancy was found in females mated during the acute phase of infection or on the 4th day of pregnancy. In the group infected on the 12th day of pregnancy, numerous complications including loss of pregnancy and stillbirths were recorded. During the acute phase of infection, parasitaemia was lower in pregnant females in comparison to infected, non-pregnant control females.

**Conclusions:**

Acute *B. microti* infection prevents the initiation of pregnancy and embryonic development if it occurs during the first trimester, and causes severe complications in foetal BALB/c mice in the second and third trimesters of pregnancy. Chronic *B. microti* infection has no detrimental impact on the initiation and development of pregnancy, but results in congenital infection of the offspring. Further study is required to determine the extent to which maternal anti-babesial immune responses contribute to compromise pregnancy in the murine model of congenital *Babesia* infection.
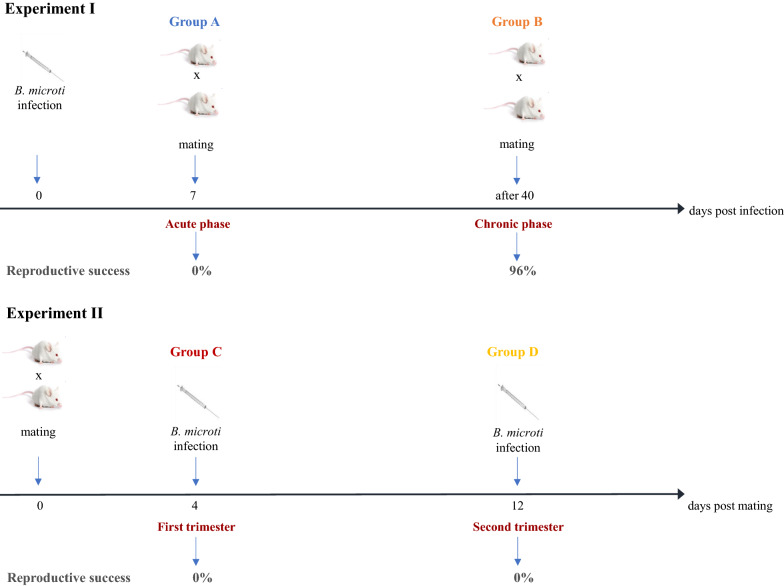

## Background

Parasitic infections may have negative effects on host breeding success and the health of offspring born to infected mothers. Congenital parasitic infection is acquired when parasites are transmitted from an infected pregnant female to her offspring, and result in an infection that persists after birth [1]. Among the best-recognised congenitally acquired parasite infections are those of *Toxocara canis* in dogs, *Toxoplasma gondii, Trypanosoma cruzi*, *Babesia microti*, and *Plasmodium* sCns [1–5].

Protozoa in the genus *Babesia* are responsible for babesiosis, a malaria-like disease in humans and animals [6]. Acute babesiosis can lead to death, but asymptomatic/subclinical infections have also been reported from humans and animals [7, 8]. Asymptomatic infections in females may have impacts on breeding and result in congenital infections in their offspring [9–11]. Congenitally acquired babesiosis has been reported for several *Babesia* spp. and has been recognised in humans [1], livestock [12–15], and dogs [16, 17]. Congenital infection has also been reported in wild rodents that serve as reservoir hosts of these parasites [9, 18], as an example in *B. microti* in mice [19], and experimental studies have supported a vertical route of transmission for *Babesia gibsoni* in dogs [20].

Reported cases of inborn babesiosis differ in their outcomes. Congenital infections in voles, *Microtus* spp., and *Peromyscus leucopus* are subclinical [9, 18], while infections in dogs result in stillbirths and/or pathology and hence severe disease [16, 17, 20]. There may also be severe consequences in livestock, as in the case of a newborn calf infected congenitally with *Babesia bovis* which died without treatment within 24 h of delivery [12].

The course of vertically transmitted babesiosis in offspring is highly dependent on the phase of the infection in mothers, and may differ between host species [19]. In our earlier work female mice with chronic infections gave birth to pups with asymptomatic, congenital infections, while those in the acute phase did not deliver any offspring [19]. In humans, 13 cases of congenital babesiosis due to *B. microti* have been reported to-date [1, 21–29]. In all cases, the symptoms of the disease (flu-like symptoms, anaemia, and hepatomegaly) in children occurred several weeks after delivery [1, 21–29]. In order to prevent and effectively treat congenital babesiosis, it is necessary to understand the mechanisms of vertical transmission (i.e. exact place, time, pathogenesis) and the consequences of parasitic infection acquired at different phases of pregnancy.

The aims of this study were (1) to evaluate and compare the success of vertical transmission of *B. microti* in female mice infected before pregnancy (mated during the acute or chronic phase of *Babesia* infection) to that of pregnant mice infected during early and advanced pregnancy; (2) to evaluate the possible influence of pregnancy on the course of parasite infections (parasitaemia); and (3) to assess and compare pathological changes in female mice and their embryos induced by parasite infections.

## Methods

### Animals

Ten- to 12-week-old BALB/c mice, 82 females (experimental and control groups) and 33 males (for mating only), were used in the course of the experiments (Table 1). All the animals were kept in standard cages provided with sawdust, nest material, food, and water *ad libitum*. Females were kept in cages in groups of 2–8 before mating. Pregnant females and dams with pups were housed in pairs. The sire males were housed individually while not paired with females, and then introduced into cages with females for one night only (1 male + 1–2 females). All of the procedures conducted on mice were approved by the First Ethics Committee for Animal Experimentation in Poland (ethical license numbers: 406/2013, 716/2015, and 536/2018), according to the principles governing experimental conditions and care of laboratory animals required by the European Union and the Polish Law on Animal Protection.

**Table 1:** Total number of females in the experimental and control groups, and number of embryos and pups recorded in the course of the experiments

Group	Experiment I		Experiment II		Control groups		
	A	B	C	D	NPI	PU	NPU
Females	6	15	20	20	9	6	6
Pregnancies	0	14	3^*,**^	20	NA	6	NA
Embryos	0	65	0^**^	77	NA	ND	NA
Pups	0	52	0	35^***^	NA	41	NA

Experiment I: females inseminated in acute (group A) and in chronic (group B) phase of *B. microti* infection. Experiment II: pregnant females infected in I (group C) and II (group D) trimester of pregnancy

*NPI* non-pregnant, infected, *PU* pregnant, uninfected, *NPU* non-pregnant, uninfected

^*^At the end of the experiment on a necropsy date

^**^In 3 females, 17 uterine scars were found, suggesting embryo implantation, but no further development

^***^None of the 35 pups born in group D survived; *NA* not applicable, *ND* not done

### *Babesia microti* strain

Female mice were infected with the *B. microti* King's 67 strain, which originated from field voles in the Oxford area, United Kingdom [30]. The parasite was passaged from infected to naive mice by intraperitoneal injections of 5 × 10^6^ infected red blood cells (iRBCs) in a volume of 0.2 ml [8, 19].

### Mating and breeding of mice

Phases of the oestrous cycle in females were identified by crystal violet staining of vaginal smears [31]. The presence of a vaginal plug in the morning, following introduction of males on the previous day, indicated that insemination had occurred. Around 8–12 days post fertilisation, females were weighed and vaginal smears were performed to determine whether pregnancy had been initiated by successful implantation. Females were housed in pairs during pregnancy. Pups developing from control group PU and from females mated during the chronic phase of infection (group B, see below: study design) were housed with their mothers until the end of the experiment.

### Study design

**Fig. 1:**
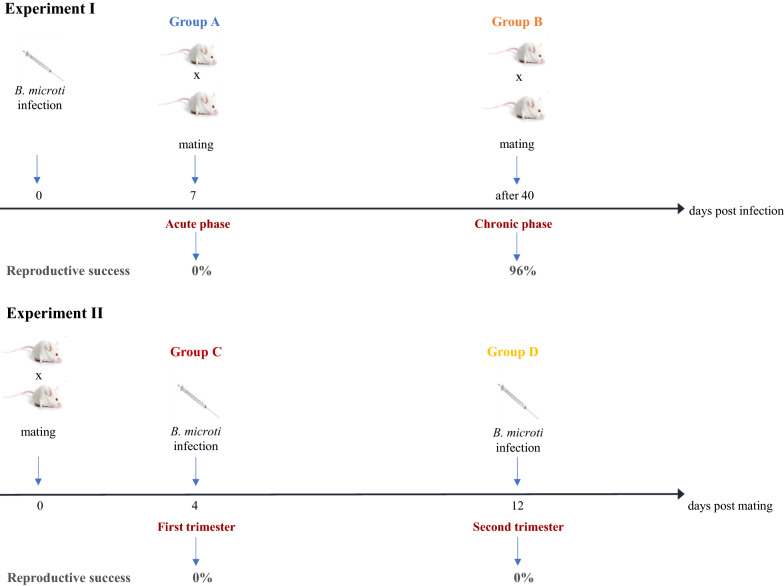
Scheme of experiments. The first set of experiments involved females mated during the acute (group A) and chronic phases of *B. microti* infection (group B). A second set of experiments involved female mice infected with *B. microti* on the 4th (group C) and 12th (group D) days of pregnancy.

The study design is presented in Fig. 1. The first set of experiments involved females infected with *B. microti* before mating. Females were assigned to two experimental groups:

In group A, six females in the appropriate phase of the reproductive cycle were mated on the 7th day post infection with *B. microti* (acute phase).

In group B, 15 females were mated during the chronic phase of *B. microti* infection, after the 40th day post infection (chronic phase).

A second set of experiments involved female mice infected with *B. microti* during pregnancy:

In group C, 20 fertilised females were infected with *B. microti* on the 4th day post mating, in early pregnancy.

In group D, 20 fertilised females were infected with *B. microti* on the 12th day post mating, in advanced pregnancy.

Three control groups of female mice were used, in total comprising 21 animals:

Control group 1 (NPI) of *B. microti* infection: nine virgin females were infected with *B. microti,* to monitor the course of infection in non-pregnant females (non-pregnant, infected).

Control group 2 of pregnancy (PU): six fertilised uninfected females were used to monitor blood parameters and the course of pregnancy in healthy females (pregnant, uninfected).

Control group 3 (NPU): six non-pregnant, uninfected females provided baseline data for healthy females in the absence of infection.

### Course of the experiments

Blood from the tip of the tail was collected for blood smears from each infected female, starting on the day of infection. From the 1st to 20th days post infection (dpi) (groups A, B, C, D, NPI), blood was collected every 2–4 days. After the 20th day post infection (group B), samples were collected every 10 days (2–3 times: on 30th, 40th, and 50th days post infection) until insemination, which took place between the 40th and 50th days post infection, depending on the phase of the oestrus cycle of each individual female.

At the end of the experiments all females were euthanised by cervical dislocation. As the weight of females from group A had not changed since fertilisation, and ultrasound examination had been unable to detect any developing embryos, females from this group were necropsied on the 12th day to check on the state of pregnancy.

Females from group B were necropsied on the 12th (1 female), 16th (3 females), and 18th (3 females) days of pregnancy, and after delivery—on the 1st (3 females), 7th (2 females), and 14th (3 females) days postpartum.

Females from group C were necropsied on the 8th (6 females), 12th (6 females), 14th (6 females), and 18–20th (2 females) days of pregnancy.

Females from group D were necropsied between the 14th and 18th days of pregnancy (2 females on 14th, 6 females on 16th, 6 females on 18th), and 6 females on the 1st day postpartum.

Six females from control group 1 (NPI, non-pregnant, infected) were necropsied on the 15th day post infection to collect samples for blood cell counts, histopathology, and evaluation of parasitaemia. Another three mice, infected with *B. microti* to compare the course of parasitaemia with females from group B, were necropsied around the 150th day post infection.

Females from control group 2 (PU, pregnant, uninfected) were necropsied on the 1st (1 females), 7th (3 females), and 14th (2 females) days postpartum.

Females from control group 3 (NPU, non-pregnant, uninfected) were necropsied at the age of 15 weeks—around the same age as females from experimental groups A, C, and D (6 females).

At necropsy of pregnant females, data on embryo development and tissue samples were collected for a range of laboratory investigations. Embryos (if present) were first collected from the uterus, washed twice in sterile water, counted and weighed individually. The course of pregnancy, defined by the appropriate size and appearance of embryos, was evaluated visually. Embryos were checked for the presence of developmental abnormalities (i.e. malformation of limbs, evidence of stillbirth/abortion).

Parameters and definitions:

“Initiation of pregnancy” was defined as successful implementation of embryos (presence of embryos or uterine scars).

“Mean litter size” was calculated as the mean number of collected pups/embryos per female in the group.

“Reproductive success” was calculated as the mean number of well-developed, normal embryos/pups per female in the group.

“Survival of offspring” was defined as the percentage of well-developed, live embryos/pups on the day of the necropsy from the total number of offspring born earlier.

“Vertical transmission” was expressed as the prevalence of *B. microti* infection (% *B. microti*-positive offspring) in the offspring of females from the experimental group, i.e. from infected mothers.

“Congenital infection” in the offspring refers to *B. microti* infection in the offspring acquired from an infected mother during the course of pregnancy.

Females from all experimental and control groups together with their offspring were necropsied. Blood samples (1 ml) were collected with a sterile syringe directly from the heart following the death of the females. Blood smears were prepared immediately for evaluation of parasitaemia (if the animal was infected), 100–200 µl of blood was kept frozen for DNA extraction, and the remaining blood sample was used for the determination of blood parameters: red blood cell (RBC) and platelets counts. Organs (brain, heart, lungs, kidneys, liver, spleen, uterus, placenta) were collected and weighed. A fragment of each organ was fixed in 10% formalin for histological examination and another fragment was frozen at a temperature of –20 °C for DNA extraction.

Uteri were washed several times in sterile water. Tools were washed in sterile water, rinsed with alcohol, and flamed to remove eventual DNA contamination. At the necropsy of offspring, blood and selected tissue samples were collected for identification of congenital *Babesia* infection. Large vessels in the foetuses/pups’ thorax were cut, and blood was collected using capillary tubes or micropipettes into spray-coated K_2_EDTA tubes (2 ml, ProfiLab, Warsaw, Poland). At each stage of embryonic development (12th, 14th, 16th, 18th days of pregnancy) we were able to collect heart and lung samples; however, on the 12th and 14th days of pregnancy, those two organs were pooled due to their small size. From the 16th day of pregnancy, these organs were collected separately, but to maintain consistency the data are presented together, i.e. pooled, for these two organs. All organ types were possible to collect postpartum, if pups were in good condition (without malformations, not eaten by the mother, etc.). Each embryo was washed several times in sterile water, as were also all isolated organs. The latter were weighed; a fragment of each organ was then frozen at a temperature of –20 °C for DNA extraction. If available, fragments of the organs were fixed in 10% formalin for histological examination.

### Laboratory methods

#### Monitoring of pregnancy development

During pregnancy females were weighed every 2–4 days. Between the 12th and 18th days of pregnancy, ultrasound examinations were performed by an experienced veterinary practitioner to assess the status of pregnancy in groups B (*n* = 11), D (*n* = 10), and control group PU (*n* = 4), which were the only groups where we recorded advanced pregnancy. The examination was carried out with a MayLab One ultrasound transducer (Esaote, Genoa, Italy), with an 18 MHz line probe at a test depth of 3–4 cm. The study was performed along both the longitudinal and transverse axes of each animal. The mice were examined without anaesthesia (to avoid any impact on the embryos), after shaving the fur on the abdomen and applying ultrasound Aquasonic^®^100 transmission gel for the ultrasound examination (Parker Laboratories, NY, USA), and were immobilised by scruffing (gripping the skin at the back of the neck). Examination was performed on the 12th, 14th, 16th, and 18th days of pregnancy. Litter size was estimated. Embryo heart rate was estimated during each monitoring session. Heart rate was measured in females and selected embryos (2–4 embryos, lying in a lateral position) from each female. The measurements were performed using a probe with a frequency of 18 MHz, using spectral Doppler, by targeting a Doppler gate (1 mm in diameter) at the heart. In the spectral Doppler, the distance between individual heartbeats was measured by manually overlaying gauges/markers (minimum of two and maximum of five spectral Doppler beat measurements during each sweep), and then the average heart beat was calculated from the recorded values.

#### Monitoring of the course of *B. microti* infection

Parasitaemia was determined on the basis of Hemacolor^®^ (Merck, Darmstadt, Germany) stained blood smears as described previously [19]. A total of 20 or 200 fields during the acute or chronic phases of infection, respectively, were inspected twice and the number of iRBCs was recorded. Parasitaemia was expressed as the percentage of infected cells.

#### Histological study and blood parameters

Histological specimens were examined as paraffin sections after staining by the haematoxylin-eosin method for evaluation of pathological changes in tissues [32]. Blood samples (0.25–0.5 ml) were collected from six females from each group into spray-coated K_2_EDTA tubes (2 ml, ProfiLab, Warsaw, Poland). RBC and platelet counts were performed by a commercial diagnostic laboratory (LabWet, Warsaw, Poland).

#### Detection of *B. microti* infection in females (experimental) and offspring (congenital)

Genomic DNA was extracted from blood and tissue samples of females, and from tissue samples of embryos and pups using a Qiagen DNeasy^®^ Blood & Tissue Kit (Qiagen, Hilden, Germany). Detection of *B. microti* was done by amplification and sequencing of the 559 bp fragment of 18S rRNA by PCR or nested-PCR (in the case of no, or weak, signal from the initial one-step PCR). The primers and thermal profiles used have been described previously [9, 19, 33]. Negative controls were performed with sterile water in the absence of template DNA. DNA of *Babesia canis* was used as positive control. PCR products were visualised following electrophoresis on 1.5% agarose gel, stained with Midori Green stain (Nippon Genetics, GmbH, Düren, Germany).

### Statistical analyses

The statistical approach has been documented comprehensively in our earlier publications [9, 19]. Analyses were carried out using SPSS v. 21.0. The Kruskal–Wallis *H* test with pairwise comparisons using Dunn's test was used to compare reproductive success and blood counts between groups. The Mann–Whitney *U* test was used to compare the following parameters: percentage of *B. microti*-positive pups and embryos, survival of offspring, mean litter size, mean body mass, and mean heart rate between two groups.

Prevalence (percentage of animals infected) and vertical transmission were analysed by maximum likelihood estimation based on log-linear analysis of contingency tables. For analysis of the prevalence of *Babesia* in pups, we fitted prevalence of *Babesia* infection as a binary factor (two levels: infected = 1, uninfected = 0), development stage (two levels: embryo = 1, pup = 2), and age of pups (three levels: 1 = 1, 7 = 2, 14 = 3 days postpartum). Prevalence of *B. microti* is presented as a percentage of mice infected with 95% confidence interval (CI_95_) calculated with bespoke software based on the tables of Rohlf and Sokal, by courtesy of F. S. Gilbert and J. M. Behnke [34].

A general linear model (GLM) was used for analysis of parasitaemia, which is reported as mean *B. microti* parasitaemia with standard error of the mean (SE). Mean parasitaemia of *B. microti* infection was calculated as the number of iRBCs/1000 RBCs. When samples were only positive by PCR, an intensity of 0.001 iRBC was implemented into quantitative statistical analyses.

## Results

### Reproductive success of *B. microti*-infected female mice

Female mice in control group PU showed no complications during pregnancy and pup delivery, with a mean litter size of 6.8 ± 1.0 (Tables 1,2). Ultrasound examination showed no malformations in embryos and all pups were delivered in good health and remained so until the day of necropsy (Table 2).

**Table 2:** Comparison of the reproductive success of females in experimental and control groups, and features of congenital *B. microti* infection in their offspring

Group	Experiment I		Experiment II		Control group
	A (*n* = 6)	B (*n* = 15)	C (*n* = 20)	D (*n* = 20)	PU (*n* = 6)
Proportion of pregnant females [Cl_95_] (*n* of pregnancies in the group/n of females in the group)	0% [0–41] (0/6)	93% [70–100] (14/15)	15% [4–37] (3/20)	100% [83–100] (20/20)	100% [92–100] (6/6)
Total *n* of offspring	0	117	17	112	41
Mean litter size ± SE (live + dead)/group	0	7.8 ± 0.9	0.9 ± 0.5	5.6 ± 0.5	6.8 ± 1.0
Alive:dead offspring	0:0	112:5	0:17	0:112	41:0
Survival -% of alive embryos/pups [Cl_95_], (infected/tested)	0% (0/0)	96% [92–98] (112/117)	0% (0/17)	0% 0/112	100% [93–100] (41/41)
Reproductive success (mean *n* of alive offspring/*n* of females)	0.0 (0/6)	7.5 ± 0.9 (117/15)	0.0 (0/17)	0.0 (0/20)	6.8 ± 1.0 (41/6)
Vertical transmission [Cl_95_], infected/tested	NA	63% [56–70] 71/112	ND	66% [59–72] 67/102	NA
Symptoms of infection in females during pregnancy	Apathy	Asymptomatic	Apathy, implantation scars in uteri, placentas in resorption	Apathy, difficulties during delivery	NA
Symptoms of infection in pups and embryos	NA	Asymptomatic	Resorption	Developmental defects, stillbirth	NA
USG observation	No visible pregnancy development	Normal size of the embryos and embryo sacs	No visible pregnancy development	Following infection, termination of pregnancy was observed (death of embryos)	Normal size of the embryos and embryo sacs

*NA* not applicable, *ND* not enough material from resorbed embryos to perform molecular examination

In Experiment I, the difference in reproductive success between groups A, B, and PU was significant (Kruskal–Wallis test, *H*_2_ = 17.52, *p* < 0.001). Pairwise comparisons using Dunn's test indicated that reproductive success in group PU was significantly higher than in group A (*p* < 0.001), but not in group B (*p* > 0.05). Reproductive success was significantly higher in group B compared to group A (*p* = 0.0001, Fig. 1). In group A, no signs of implantation of embryos were observed during necropsy (Table 2). The proportion of pregnant females and reproductive success in group B were similar to the results in the control group PU (Tables 1,2, not significant, NS). Females in group B presented no evidence of complications during pregnancy; their mean body weight on the 18th day of pregnancy was similar to the mean weight of the females in the control group PU: 34.85 ± 4.01 g and 36.66 ± 0.88 g, respectively (NS). Ultrasound examination showed normal pregnancy development; growth of embryos and their heart rates (beats/minute) were normal, and there were no significant differences in comparison to embryos of females from control group PU (205.96 ± 10.38/47.44 ± 4.84 vs 195.25 ± 13.15/47.23±5.14 on the 18th day of pregnancy, respectively).

The mean litter size was similar in groups B and PU (7.8 ± 0.9 vs 6.8 ± 1.0, respectively; NS, Table 2). Survival of offspring was 96% in group B (two embryos were found reabsorbed in the uterus of a female necropsied on the 18th day of pregnancy and three pups were killed by the dam between the 2nd and 4th days postpartum). Pups were asymptomatic and in good condition, no malformations were observed. Survival was similar to the survival of the offspring in the control group PU (Table 2). At the 14th day postpartum, the mean weight of pups from infected females from group B was similar to the mean weight of pups from uninfected females from control group PU (6.9 ± 0.2 g vs 6.8 ± 0.2 g respectively; NS).

In group C, infection with *B. microti* resulted in a total lack of embryos. No embryos (only uterine scars) were recorded during necropsy (undertaken between the 10th and 12th days of expected pregnancies). Uterine scars were found in only three females (15%) from this group, suggesting the implantation of 17 embryos in total.

In group D, females gained weight and showed no signs of complications until the 12th day of pregnancy (on the day of infection). The development of pregnancies until the 12th day of pregnancy was normal (living embryos recorded during ultrasound monitoring in all females). The mean heart rate of embryos was similar to the mean heart rate of embryos from the control group PU (204.66 ± 7.44/39.36 ± 4.82 and 197.04 ± 12.71/48.98 ± 4.62*,* respectively, NS). However, numerous complications appeared after infection with *B. microti* (Table 2). Ultrasound examination showed that embryos that were alive on the day of infection died on consecutive days post infection (between the 2nd and 6th days post infection). The embryos’ hearts stopped beating and females had problems with delivery—pups were stillborn or died shortly after birth. As no living offspring were obtained from experimental groups C and D, females had no reproductive success (0% of live offspring) in comparison to the control group PU (100%; Fig. 1, Table 2).

On the 1st day postpartum, the combined mean weight of dead and live pups from group D was half the mean weight of the live pups from the control group PU (0.62 ± 0.08g vs 1.31 ± 0.13g; Mann–Whitney *U* test, *U*_13,13_ = 142.0, *p* < 0.05). A number of visible malformations were recorded in embryos during necropsy of eight females from group D—limbs or heads of the embryos were in the process of reabsorption.

### Vertical transmission of *B. microti*

Overall vertical transmission in group B was 63% [56–70%] (Table 2). In group B, *B. microti* DNA was detected in 58% [Cl95: 46–69%] of embryos and 73% [57–86%] of pups (NS).

Interestingly, older pups showed significantly higher prevalence of infection than younger pups (*Babesia* infection × day postpartum: *χ*^2^ = 9.75, *df* = 2, *p* < 0.05). During the necropsies performed on the 1st day postpartum, 43% [21–68%] of tested pups were *B. microti*-positive; however, prevalence was higher on the 7th (88% [50–99%]) and 14th days postpartum (90% [68–98%]). There was no sex difference in the presence of congenital infection in pups—71% [46–88%] of males and 84% [68–98%] of females tested positive (NS).

The detection of *B. microti* DNA in tissues of offspring and placentas from experimental groups B and D is shown in Table 3 (description further in the text). No clinical signs of the acute phase of the *Babesia* infection (dark-coloured urine, febrile seizures, anorexia, apathy) were noted among the infected pups from group B (Table 2).

**Table 3:** Occurrence of *B. microti* DNA in the tissue of embryos and pups, and placentas from experimental groups B and D.

Specimen tested	Group B		Group D		Statistical values	*p* values
	% [95% Cl]	Positive/tested	% [95% Cl]	Positive/tested		
Blood	63% [56–70]	71/112	66% [59–72]	67/102	NS	*p * > 0.05
Brain	15% [6–21]	6/41	7% [0–30]	1/15	NS	*p * > 0.05
Heart and lungs	63% [56–70]	71/112	63% [50–75]	59/93	NS	*p * > 0.05
Liver	13% [7–23]	9/69	53% [29–78]	8/15	*U =* 309.0	*p * < 0.05
Spleen	16% [7–32	7/44	63% [29–89]	5/8	*U* = 94.0	*p * < 0.05
Kidneys	13% [5–29]	6/45	60% [33–81]	9/15	*U =* 180.0	*p * < 0.05
Placenta	69% [53–82]	29/42	98% [93–100]	57/58	*U =* 876.0	*p * < 0.001

*NS* not significant

The DNA of *B. microti* was detected in 66% [59–72%] of tested offspring (67 positive/102 tested): 49% [38–61%] (34 positive/69 tested) in embryos and 100% [92–100%] (33/33) in examined pups (Mann–Whitney *U* test, *U*_69,33_ = 561.0, *p* < 0.001, Table 3) from group D. The tissues of six embryos were in too poor condition to enable DNA extraction. As explained earlier, pups in this group were mostly stillborn, with visible head and/or limb malformations, or died a few hours after delivery.

There was no significant difference in the prevalence of infection between embryos from groups B and D (58% [46–69%] vs 49% [38–61%], respectively, NS). There was no significant difference in the detection of *Babesia* DNA in embryos necropsied during the second (on 12th and 14th days of pregnancy) and third (on 16th and 18th days of pregnancy) trimesters of pregnancy. The percentage of infected pups in group D was higher than in group B on the 1st day postpartum (100% vs 43%, respectively, Mann–Whitney *U* test, *U*_33,14_ = 120.0, *p* < 0.001).

*Babesia* DNA was found in all types of tested organs (Table 3). A significantly higher percentage of positive samples was noted in the livers, spleens, kidneys and placentas of offspring from group D in comparison to group B (Table 3).

### Comparison of the course of *B. microti* infection in female mice

**Fig 2:**
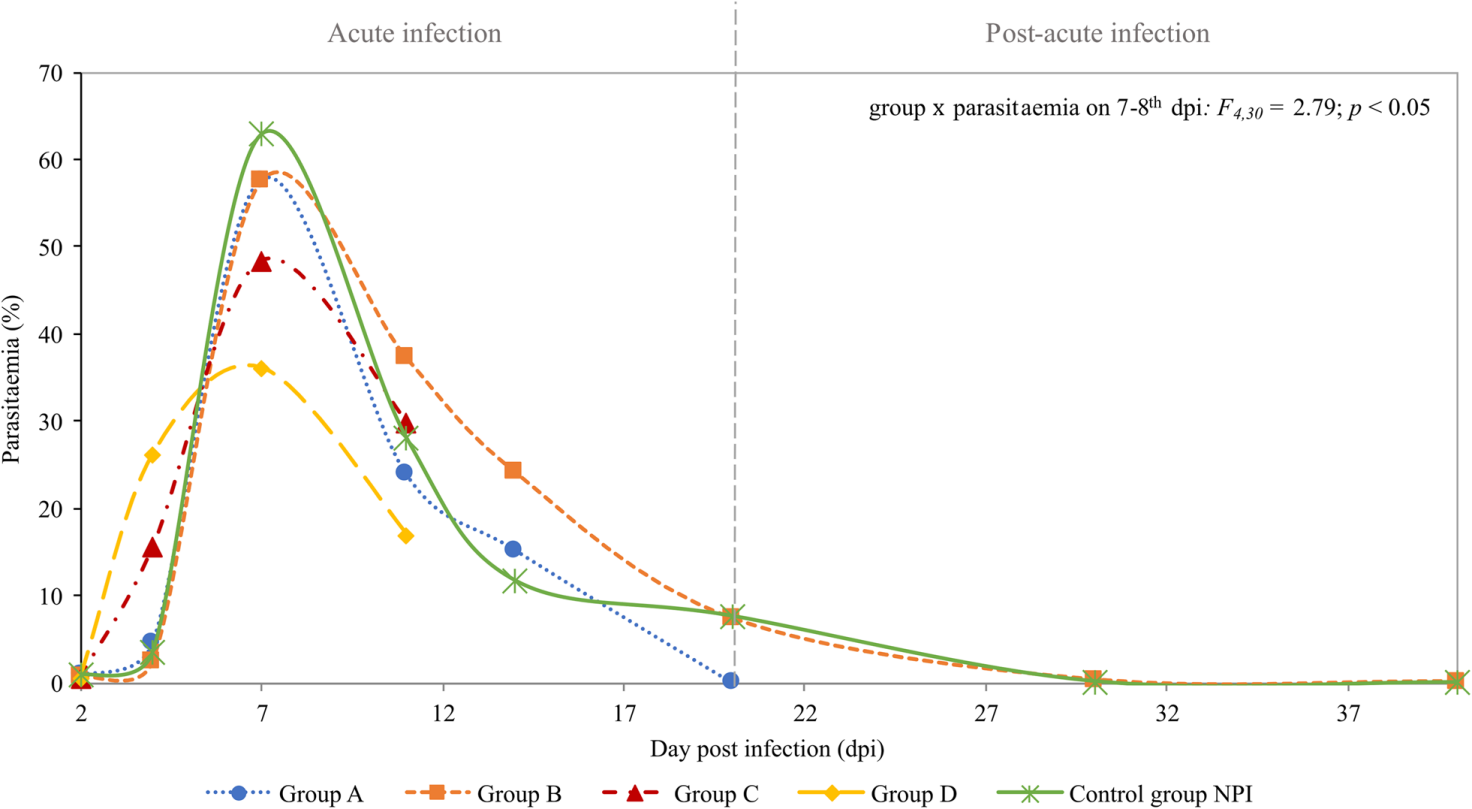
The course of *B. microti* infection in experimental and control groups. Comparison of the course of *B. microti* infection (Cl_95_) in mice pregnant in the different phases of the parasite infection. Level of *B. microti* parasitaemia on the selected day post infection. Parasitaemia is shown as a percentage of iRBCs found in mouse peripheral blood, measured on the Giemsa-stained thin smears. Data points are the mean for three to six animals.

A comparison of the courses of *B. microti* infection during the acute and post-acute phases of infection in the experimental groups is presented in Fig. 2. In all experimental groups, the acute phase of *B. microti* infection was reflected in high parasitaemia during the first two weeks of infection. The highest parasitaemia was observed on the 7–8th dpi in all experimental and control groups (Fig. 2).

Parasitaemia on the 7–8th dpi was highest in control group NPI, lower in groups A and B, and lowest in groups C and D (group × parasitaemia on 7–8th dpi*: F*_4,30_*=* 2.79; *p* < 0.05; Fig. 2). Among experimental groups, maximum parasitaemia during the acute phase of infection was slightly higher in non-pregnant females in comparison to females with confirmed pregnancy (55.4% vs 40.5%, respectively; pregnancy × parasitaemia: *F*_4,42_* =* 7.97, *p* < 0.05).

No significant associations were observed between parasitaemia and success of vertical transmission or between parasitaemia and reproductive success of a female across the experimental groups.

### Molecular detection of *B. microti* DNA in blood and tissue of females and dams

Statistical evaluation of the differences between treatment groups in detection of *B. microti* DNA in blood and other tissues of females is presented in Table 4. The DNA of *B. microti* was found in blood samples collected from all infected females in experimental groups and from control group NPI (non-pregnant, infected). *Babesia* DNA was identified in all tested organs (Table 4). The highest percentage of *B. microti*-positive tissues was detected in hearts and lungs, with the lowest in uteri.

**Table 4:** Occurrence of *B. microti* DNA [%; Cl_95_] in blood and organs of BALB/c females.

Specimen tested/sample	Group A (*n* = 6)	Group B (*n* = 15)	Group C (*n* = 20)	Group D (*n* = 20)	Control group NPI (*n* = 9)	Statistical values	*p* values
Blood	100% [59–100]	100% [27–94]	100% [83–100%)	100% [83–100]	100% [59–100]	NS	*p *> 0.05
Brain	67% [27–94]	53% [29–78]	70% [48–86]	100% [83–100]	100% [59–100]	*χ*^ 2^ = 3.97, *df* = 4	*p * < 0.05
Heart	100% [59–100]	53% [29–78]	70% [48–86]	100% [83–100]	100% [59–100]	*χ*^ 2^ = 4.97, *df* = 4	*p * < 0.05
Lungs	67% [27–94]	60% [33–81]	85% [63–96]	100% [83–100]	100% [59–100]	*χ*^ 2^ = 3.54, *df* = 4	*p * < 0.05
Liver	67% [27–94]	27% [10–53]	35% [17–58]	90% [68–98]	100% [59–100]	*χ*^ 2^* =* 8.29, *df* = 4	*p * < 0.001
Spleen	67% [27–94]	40% [19–67]	60% [37–79]	100% [83–100]	100% [59–100]	*χ*^ 2^* =* 6.18, *df* = 4	*p * < 0.001
Kidneys	67% [27–94]	47% [22–71]	55% [32–76]	90% [68–98]	100% [59–100]	*χ*^ 2^* =* 14.43, *df* = 4	*p * < 0.05
Uterus	0% [0–41]	13% [2–40]	25% [10–48]	70% [48–86]	100% [68–100]	*χ*^ 2^*=* 10.30, *df* = 4	*p * < 0.001

*NS* not significant

### Histopathological changes in female BALB/c mice

Material for histological examination was collected from selected females in experimental and control groups, and a variety of histopathological changes (lesions) were found in the organs of both experimental and control groups (Table 5). There was no significant effect of *Babesia* infection on the frequency of lesions in the organs of the mothers. Some of the detected changes were associated with aging or development of pregnancy (results not shown).

**Table 5:** Occurrence of pathological changes in organs and organ tissues of females from experimental groups.

Organ type	Pathological changes	Group A	Group B	Group C	Group D	Control group PU	Control group NPU	Statistical values	*p* value
Spleen	Extramedullary haematopoiesis	83% (5/6)	83% (5/6)	59% (10/17)	93% (14/15)	100% (4/4)	75% (3/4)	NS	*p * > 0.05
	Lymphoid hyperplasia	50% (3/6)	50% (3/6)	76% (13/17)	40% (6/15)	50% (2/4)	50% (2/4)	NS	*p* > 0.05
	Presence of siderophages	50% (3/6)	83% (5/6)	6% (1/17)	47% (7/15)	0% (0/4)	50% (2/4)	*χ*^2^ = 17.32, *df* = 5	*p * < 0.05
	Congestion	0% (0/6)	0% (0/6)	18% (3/17)	7% (1/15)	0% (0/4)	0% (0/4)	NS	*p* > 0.05
Liver	Inflammation	100% (6/6)	83% (5/6)	100% (6/6)	93% (14/15)	100% (5/5)	75% (3/4)	NS	*p* > 0.05
	Degeneration of the hepatocytes	0% (0/6)	33% (2/6)	0% (0/17)	47% (7/15)	0% (0/5)	0% (0/4)	NS	*p* > 0.05
	Hyperaemia	0% (0/6)	67% (4/6)	6% (1/17)	47% (7/15)	80% (4/5)	50% (2/4)	*χ*^2^ = 21.40, *df* = 5	*p * < 0.05
	Necrosis of the hepatocytes	50% (3/6)	0% (0/6)	24% (4/17)	53% (8/15)	20% (1/5)	25% (1/4)	NS	*p* > 0.05
	Cholestasis in hepatocytes	0% (0/6)	83% (5/6)	12% (2/17)	13% (2/15)	0% (0/5)	50% (2/4)	*χ*^2^ = 27.72, *df* = 5	*p *< 0.001
Kidneys	Necrosis of rental tubules	83% (5/6)	100% (6/6)	94% (16/17)	87% (13/15)	67% (4/6)	100% (4/4)	NS	*p* > 0.05
	Haemoglobin in renal tubule cells	0% (0/6)	17% (1/6)	12% (2/17	20% (3/15)	0% (0/6)	25% (1/4)	NS	*p* > 0.05
	Acute nephritis	0% (0/6)	33% (2/6)	0% (0/17)	0% (0/15)	0% (0/6)	0% (0/4)	NS	*p* > 0.05
	Glomerulonephritis	33% (2/6)	0% (0/6)	6% (1/17))	0% (0/15)	17% (1/6)	25% (1/4)	NS	*p* > 0.05
	Blood haemorrhages	0% (0/6)	17% (1/6)	0% (0/17)	27% (4/15)	33% (2/6)	0% (0/4)	NS	*p* > 0.05
	Hyperaemia	17% (1/6)	50% (3/6)	24% (4/17)	40% (6/15)	67% (4/6)	0% (0/4)	NS	*p* > 0.05
	Proteinuria	0% (0/6)	0% (0/6)	12% (2/17)	0% (0/15)	0% (0/6)	0% (0/4)	NS	*p* > 0.05
	Parenchymatous degeneration of rental tubules cells	33% (2/6)	67% (4/6)	76% (13/17)	80% (12/15)	100% (6/6)	0% (0/4)	*χ*^2^= 18.43, *df *= 5	*p *< 0.05
Brain	Cerebral oedema	83% (5/6)	83% (5/6)	69% (11/16)	77% (10/13)	100% (6/6)	50% (2/4)	*χ*^2^= 11.47, *df* = 5	*p* < 0.05
	Neuronophagia	33% (2/6)	0% (0/6)	50% (8/16)	0% (0/13)	50% (3/6)	50% (2/4)	*χ*^2^= 18.11, *df* = 5	*p* < 0.05
	Nerve cell (neuron) necrosis	33% (2/6)	67% (4/6)	56% (9/16)	69% (9/13)	50% (3/6)	50% (2/4)	NS	*p* > 0.05
	Nerve cells degeneration	33% (2/6)	17% (1/6)	6% (1/16)	46% (6/13)	0% (0/6)	25% (1/4)	*χ*^2^= 16.08, *df* = 5	*p* < 0.05
	Hyperaemia	0% (0/6)	33% (2/6)	0% (0/16)	0% (0/13)	33% (2/6)	0% (0/4)	NS	*p* > 0.05
	Proliferation of microglia	0% (0/6)	0% (0/6)	13% (2/16)	15% (2/13)	0% (0/6)	25% (1/4)	NS	*p* > 0.05
	Inflammation	0% (0/6)	0% (0/6)	6% (1/16)	0% (0/13)	0% (0/6)	0% (0/4)	NS	*p* > 0.05
Heart	Necrosis of cardiomyocytes	67% (4/6)	17% (1/6)	76% (13/17)	20% (3/15)	33% (2/6)	50% (2/4)	*χ*^2^= 14.77, *df* = 5	*p* < 0.05
	Inflammation of the heart muscle	50% (3/6)	33% (2/6)	41% (7/17)	27% (4/15)	50% (3/6)	25% (1/4)	NS	*p* > 0.05
	Focal fibrosis of myocardium	0% (0/6)	0% (0/6)	6% (1/17)	0% (0/15)	0% (0/6)	0% (0/4)	NS	*p* > 0.05
	Congestion of the heart muscle	0% (0/6)	50% (3/6)	6% (1/17)	20% (3/15)	83% (5/6)	0% (0/4)	*χ*^2^ = 20.87, *df* = 5	*p* < 0.05
	Atrophy of myocardium	0% (0/6)	0% (0/6)	18% (3/17)	0% (0/15)	0% (0/6)	25% (1/4)	NS	*p* > 0.05
	Hyaline degeneration myocardium	0% (0/6)	0% (0/6)	12% (2/17)	0% (0/15)	0% (0/6)	0% (0/4)	NS	*p* > 0.05
Lungs	Atelectasis	50% (3/6)	100% (6/6	76% (13/17)	73% (11/15)	100% (6/6)	0% (0/4)	*χ*^2^ = 29.08, *df* = 5	*p* < 0.001
	Oedema	0% (0/6)	0% (0/6)	6% (1/17)	0% (0/15)	0% (0/6)	0% (0/4)	NS	*p* > 0.05
	Emphysema	100% (6/6)	67% (4/6)	53% (9/17)	40% (6/15)	83% (5/6)	100% (4/4)	*χ*^2^ = 14.45, *df* = 5	*p* < 0.05
	Congestion	0% (0/6)	50% (3/6)	29% (5/17)	20% (3/15)	67% (4/6)	25% (1/4)	NS	*p* > 0.05
	Necrosis	17% (1/6)	0% (0/6)	0% (0/17)	7% (1/15)	0% (0/6)	0% (0/4)	NS	*p* > 0.05
	Siderophages	0% (0/6)	17% (1/6)	6% (1/17)	0% (0/15)	17% (1/6)	0% (0/4)	NS	*p* > 0.05
	Pneumonitis, acute interstitial inflammation	0% (0/6)	17% (1/6)	94% (16/17)	40% (6/15)	67% (4/6)	0% (0/4)	NS	*p* > 0.05
Uterus	Blood haemorrhages in wall and mucous membrane	0% (0/3)	0% (0/6)	0% (0/16)	36% (5/14)	25% (1/4)	0% (0/4)	NS	*p* > 0.05
	Hyperaemia of wall and mucous membrane	0% (0/3)	50% (3/6)	0% (0/16)	21% (3/14)	25% (1/4)	0% (0/4)	*χ*^2^ = 13.29, *df *= 5	*p* < 0.05
	Inflammation of the mucous membrane	0% (0/3)	50% (3/6)	69% (11/16)	43% (6/14)	25% (1/4)	25% (1/4)	NS	*p* > 0.05
	Necrosis	0% (0/3)	0% (0/6)	0% (0/16)	7% (1/14)	0% (0/4)	0% (0/4)	NS	*p* > 0.05
	Siderophages in myometrium	0% (0/3)	33% (2/6)	6% (1/16)	0% (0/14)	0% (0/4)	0% (0/4)	NS	*p* > 0.05
	Oedema of mucous membrane	0% (0/3)	0% (0/6)	0% (0/16)	7% (1/14)	0% (0/4)	0% (0/4)	NS	*p* > 0.05

*NS* not significant

### Histopathological changes in offspring

Material for histological examination was collected from selected pups in experimental group B and control group PU on the 14th day postpartum. A variety of histopathological changes were found in the organs of pups from group B as well as in pups from control group PU (Table 6). There was no significant effect of *B. microti* infection on the occurrence of lesions in the organs of pups. Atrophy of cardiac myocytes occurred more frequently in heart tissues in which *B. microti* DNA was detected in comparison to negative samples (4/6 = 67% vs 1/11 = 9%; *χ*^ 2^ = 6.75, *df* = 1, *p <* 0.05), but no other statistical associations were found between the presence of infection (molecular detection) and pathological changes.

**Table 6:** Occurrence of pathological changes in tissues of pups from group B and control group PU on 14th day postpartum.

Organ	Type of pathological changes	group B (*n* = 18)	Control group PU (*n* = 12)	Statistical values
Spleen	Extramedullary haematopoiesis	93% (13/14)	91% (10/11)	NS
	Lymphoid hyperplasia	57% (8/14)	91% (10/11)	NS
Liver	Inflammation	100% (18/18)	100% (12/12)	NS
	Congestion	83% (15/18)	50% (6/12)	NS
	Degeneration of the hepatocytes	6% (1/18)	8% (1/12)	NS
	Extramedullary haematopoiesis	50% (9/18)	42% (5/12)	NS
Kidneys	Necrosis of rental tubules epithelium	100% (18/18)	92% (11/12)	NS
	Congestion	61% (11/18)	50% (6/12)	NS
Brain	Cerebral oedema	94% (17/18)	100% (12/12)	NS
	Neuronophagia	6% (1/18)	25% (3/12)	NS
	Nerve cell (neuron) necrosis	28% (5/18)	42% (5/12)	NS
	Hyperaemia	22% (4/18)	17% (2/12)	NS
Heart	Necrosis of cardiomyocytes	44% (8/18)	83% (10/12)	*χ*^2^ = 4.84, *df* = 1, *p < *0.05
	Inflammation of the heart muscle	61% (11/18)	67% (8/12)	NS
	Atrophy of cardiac myocytes	28% (5/18)	8% (1/12)	NS
	Congestion of the heart muscle	22% (4/18)	42% (5/12)	NS
Lungs	Atelectasis	100% (17/17)	91% (10/11)	NS
	Pneumonitis, interstitial inflammation	88% (15/17)	91% (10/11)	NS
	Emphysema	29% (5/17)	18% (2/11)	NS
	Congestion	47% (8/17)	45% (5/11)	NS
	Siderophages	6% (1/17)	0% (0/11)	NS

### Blood parameters

**Fig 3:**
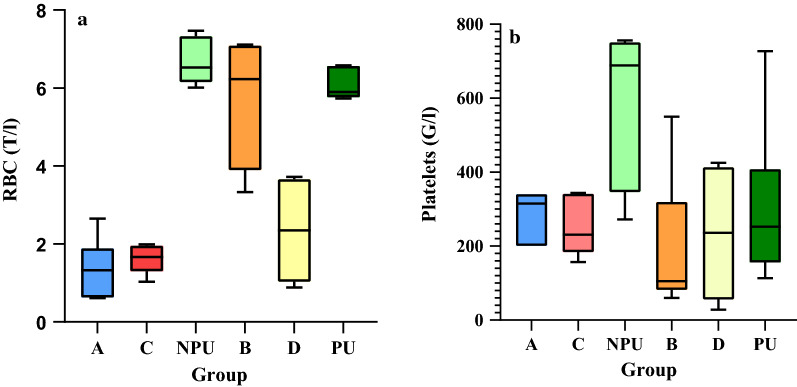
Comparison of the blood count results. **a** RBCs and **b** platelets in females mated on the 7th day post infection (group A), infected on the 4th day post mating (group C), in control group of infected, non-pregnant, uninfected females (NPU); in females mated after the 40th day post infection (group B), infected on the 12th day post mating (group D), and in control group of pregnant, uninfected females (UI). Whiskers indicate the lowest (lower) and the highest (upper) values.

As females from groups A and C presented no evidence of pregnancy, mean counts of RBCs and platelets from these groups were compared to the counts obtained from group NPU (non-pregnant, uninfected females) (Fig. 3a, b).

There was a significant difference in the RBC count between tested groups (Kruskal–Wallis test, *H*_2_ = 11.79; *p* < 0.05). Pairwise comparisons using Dunn's test indicated that the mean RBC count in group NPU was observed to be significantly higher from those of group A (*p* < 0.05) and group C (*p* < 0.05). No other significant differences were observed between these groups.

There was a significant difference in the platelet count between tested groups A, C and NPU (Kruskal–Wallis test, *H*_2_ = 8.26; *p* < 0.05). Pairwise comparisons using Dunn's test indicated that platelet counts in group NPU were observed to be significantly higher than in group C (*p* < 0.05) but not group A (NS). No other significant differences were observed between these groups.

Mean blood counts of females in groups B and D were compared to the counts from group PU (pregnant, uninfected females) (Fig. 3a, b). There was a significant difference in the RBC count between tested groups (Kruskal–Wallis test, *H*_2_ = 9.23; *p* < 0.05). The mean RBC count was significantly higher in group B in comparison to group D (*p* < 0.05). The mean RBC count (but not the platelet count) in the control group PU was twice as high as that in group D (*p* < 0.05, Fig. 3a). No other significant differences were observed between these groups.

No significant difference in the platelets count was found between groups B, D, and PU (Kruskal–Wallis test, *H*_*2*_ = 1.56; *p* = 0.459).

## Discussion

Our experiments confirmed the negative influence of acute *Babesia* infection on the development of pregnancy and the reproductive success of BALB/c mice. In this study, female mice that were inseminated during the acute phase of *B. microti* infection or shortly before implantation of embryos did not become pregnant. Infection during the second trimester of pregnancy also had a negative influence on the course of pregnancy, with stillbirth or death of pups shortly after delivery. However, in pregnancies that were initiated during the chronic phase of infection, infections had no detectable effect on the development of embryos or success of pregnancy, but were associated with a high degree of vertical transmission of *Babesia* to the offspring.

When female mice were infected shortly before embryo implantation (groups A and C), either pregnancies failed to develop or the embryos were reabsorbed (presence of scars) in the first few days post insemination. We also observed a marked negative influence of *B. microti* infection on the development/outcome of more advanced pregnancies. Mice infected during the second trimester delivered infected pups (group D), which were unable to survive longer than one day post delivery, or pregnancy was terminated before delivery, as reflected in signs of embryo reabsorption at necropsy. This marked detrimental impact of infection on the initiation and course of pregnancy might be the consequence of intense parasitaemia and the resulting significant reduction in maternal RBCs and platelets, leading to severe anaemia and thrombocytopenia. *Babesia microti* infection causes a reduction in RBC counts because of both sequestration of infected cells and disintegration of the iRBCs following emergence of merozoites after intra-erythrocytic reproduction [35]. Acute babesiosis in women also leads to severe anaemia and thrombocytopenia if diagnosed in the second or third trimesters [36, 37]. Anaemia is a prominent symptom of babesiosis, and this was observed in females from groups A, C, and D during the initial and acute phases of infection (between 2nd and 20th days post infection). Advanced anaemia may play a role in the observed low reproductive success in experimental groups (i.e. because of limited oxygen transport to the developing foetuses). Our results have some similarity to those of experimental infections with *Plasmodium berghei* and *Plasmodium chabaudi* AS in pregnant female mice, which resulted in low birth weights of pups, an increased frequency of abortions, and greatly increased foetal death rates [38, 39]. The marked detrimental impact of *B. microti* infection on the initiation, development, and survival of pregnancies is consistent with the reported high mortality in children with congenital parasitic infections, mortality occurring between the 1st and 30th days after birth if not treated for congenital toxoplasmosis, Chagas disease, and malaria [38, 40, 41]. Similarly, in nonimmune pregnant women, malarial infection during the first or second trimester has been associated with frequent occurrence of spontaneous abortion [42]. *Plasmodium vivax* infections are also associated with an increased likelihood of miscarriage, the occurrence of intrauterine growth restriction [43], and lowered birth weight [44]. The results presented in the current study, in which females mated on the day of infection with *B. microti* did not deliver pups, are consistent with these findings [19].

In this study, the reproductive success of chronically infected females was much higher than in our previous study [19] and in a laboratory study of vertical transmission of *B. microti* in *P. leucopus*. In the latter, 50% of female mice acquired *B. microti* infection via tick bites, and from these six infected individuals only three females produced offspring [10]. *P. leucopus* mice are a natural reservoir host of *B. microti*, which might be the reason that this species does not experience the same side effects and birth defects in the offspring as in the inbred *Mus musculus* BALB/c strain. To some extent our results may be the outcome of a different approach in study design in comparison to our previous experiments. More thorough cytological examination in the current study, in comparison to our previous work, enabled us to increase the reproductive success by mating females exactly at the oestrus stage [19]. In our previous study, female reproductive status was determined by visual assessment of the vaginal opening, and when assessed to be optimal was followed by introduction of male mice for a few consecutive nights [19]. The procedural changes implemented in the current study may have increased the proportion of females that became pregnant. In the mice that became pregnant during chronic babesiosis (group B), we failed to observe any abnormalities in the reproductive success or in the condition of offspring. Pups delivered by chronically infected females presented no manifestations of worsened condition (their heartbeat rate during pregnancy and birth weight were normal) in the current study, but also in our previous experiments, where only female mice in the post-acute and chronic phase of infection gave birth to viable offspring [19]. In another study, pups delivered by *B*. *microti*-infected *P. leucopus* females were born in good condition, and were able to reproduce and pass *B. microti* infection to the next generation of mice [10].

Parasites are known to employ a spectrum of evasive measures to avoid host immunity even when the host’s immune function is intact [11, 45, 46], and thus by facilitating persistent infection increase the probability of transmission. For example, the invasion of RBCs limits pathogen exposure. Expression of *Babesia*-induced adhesion molecules on the RBC surface causes adherence of parasite-infected erythrocytes to the vascular endothelium to avoid destruction in the spleen [11, 45–47]. As a result, an almost symbiotic relationship is created between the host’s immune system and the parasite, allowing the development of persistent infection [45]. On the other hand, chronic (“asymptomatic”) malaria has severe consequences for mothers and their newborns. While women with placental malaria have no obvious clinical signs of malaria infection during their pregnancy, there may be severe consequences at parturition and for survival of the neonates. Circulating chronic infections are a major source of placental infection, associated with placental inflammation, fibrosis, and functional insufficiency, leading directly to miscarriage, preterm delivery, low birth weight, and peripartum haemorrhage and, thus, increased maternal and neonatal mortality [48–51].

Our previous study revealed that the success of vertical transmission was dependent on the phase of the infection in pregnant mice [19]. Since the circulation between the female and the foetus forms only on day 9 or 10 of gestation in mice, vertical transmission of parasites is not expected to occur earlier, ie. in the first trimester [1, 52]. It is possible that placental breaches/tears resulting from damage induced by placental inflammatory responses or appearing naturally close to the end of pregnancy, particularly during parturition, can facilitate congenital transmission of parasites from maternal blood [1]. The success of vertical transmission of *B. microti* in the current study was markedly lower than in our previous experiments [19]. This difference between studies may be linked to the time of blood collection and changes in maternal and individual immunity in pups. In the current experiment, blood samples were taken between the 12th day of pregnancy and 14th day postpartum. In our pervious experiments, blood samples were taken after the 28th day postpartum [19]. Interestingly, human infants with congenital babesiosis develop symptoms (fever, fatigue, irritability, and decreased oral intake) and are admitted to hospital following 4-6 weeks postpartum [26, 53]. A similar delay in the appearance of clinical signs of babesiosis has been observed in pups with congenital infections of *B. canis—*at 6 weeks, a period of life that is believed to be crucial in the development of immunity in dogs [17]. During this period, maternally derived immunity decreases rapidly, to enable the development of individual immunity in the offspring [54]. It is possible that in the mouse model, an impaired maternal immune response gives *B. microti* an opportunity to multiply if individual immunity is not sufficiently intense.

Wild rodents that serve as hosts for *B. microti* usually present with very low parasitaemia (0.0001–0.004% RBCs), characteristic of chronic infections [9, 55, 56]. The course of parasitaemia in all the experimental groups was typical of *B. microti* infections in BALB/c mice, with a peak of parasitaemia between the 6th and 8th days post infection and an apparent clearance of circuiting iRBCs by the 30th to 40th days post infection [8, 19]. However this is not typical for reservoir host species in the wild [56]. Moreover, molecular examination in our study has provided evidence for prolonged low-intensity persistent infections even after blood smears were no longer detecting iRBCs. These results may support the hypothesis of Bednarska et al. [19], in which it was proposed that *B. microti* modulates the immune system, and when chronic infection is established and well stabilised in mice, the hormonal changes associated with the development of pregnancy do not affect or alter the course of infection. Further investigations, focusing on immunological and hormonal responses to *B. microti* infection during pregnancy, are required to fully clarify these relationships.

In our model, pregnancy and lactation during the chronic phase of infection did not change the level of parasitaemia significantly. It has been observed elsewhere that some pregnant and lactating females in the post-acute phase of *B. microti* infection show minor increases in parasitaemia [19], but such modulation of the course of infection was not recorded in the current study. Haemoprotozoan parasitaemia levels in pregnant women/females appear to be an important factor contributing to success of vertical transmission, e.g. congenital infections with *T. gondii*, *T. cruzi,* and *P. falciparum* occur more frequently in mothers displaying high levels of parasitaemia [1]. However, in our study there was no evidence to support the idea that the intensity of parasitaemia is a crucial factor in the success of vertical transmission or reproductive success of infected mice.

The success of vertical transmission of *B. microti* in mice was high, regardless of the phase of infection in females. In other studies, vertically transmitted *B. microti* infection was detected in 81% of embryos of infected female voles [9] and in 74% of embryos and pups collected from infected *P. leucopus* [10, 18], and 96% of pups delivered by infected mice were also infected [19]. In *P. leucopus, B. microti* infection lasted for over a year [10], and vertical transmission was possible for 11 months after infection of the females. Vertically infected *P. leucopus* females gave birth to offspring in which the prevalence of vertically acquired *B. microti* infection was 38%. Despite the high efficiency of vertical transmission, vector-mediated transmission is still required for long-term maintenance of *B. microti* in natural populations, because prevalence in offspring decreases over time [10]. It has been shown that vertically infected mice serve as a source of horizontal transmission to a feeding tick vector (61% feeding larvae and 58% of nymphs), and the infected ticks were able to infect susceptible hosts [10]. We know that at least until the 50th day post infection, BALB/c mice may give birth to vertically infected offspring. In this study, only the offspring from experimental group B could serve as a source of horizontal transmission to a feeding tick vector or for further studies of the efficiency of vertical transmission.

The DNA of *B. microti* was detected in all the tested organ tissues of females and their offspring. The highest percentage of *B. microti*-positive tissues in females was the placentas, hearts, and lungs, with the lowest percentage in the uteri, and in pups and embryos from groups B and D. The highest percentage of *Babesia*-infected tissues was spleen, heart, and lungs. There was no difference in the detection of *B. microti* DNA in blood, brains, hearts, and lungs of pups and embryos from experimental groups B and D. These results suggest that the level of parasitaemia in mice does not influence the success of vertical transmission. Our study demonstrated that if the female is infected, the probability of successful transmission is high. The high frequency of parasite DNA detection in lungs and hearts of offspring from both groups may be associated with sequestration of iRBCs in lung capillaries, which has been observed previously during *Babesia* spp. infections [57]. The occurrence of cerebral infection was rare, regardless of the phase of maternal infection. In the case of liver, spleen, kidneys and also placentas, DNA detection was higher in offspring of females with a higher intensity of *B. microti* infection compared to the offspring of females in the chronic phase of infection, with parasitaemia close to zero. Despite these observations, no significant influence of the mother’s parasitaemia on the success of vertical transmission was found. Sequestration of iRBCs in the spleens, lungs, and hearts has been described in bovines infected with *B. bovis*, and hamsters experimentally infected with *Babesia* WA1 [58, 59]. High parasitaemia in placental blood, while lower in neonates, has been observed in malarial infections [4]. Poovassery and Moore described the accumulation of *P. chabaudi* AS-infected erythrocytes in the placentas of infected mice as a manifestation of specific placental sequestration [39]. It is possible that placental accumulation is a phenomenon also observed in *Babesia*-infected placental tissues, and future investigations will be necessary to elucidate the exact role of placental parasitaemia in congenital babesiosis.

The geographic range and the number of recorded cases of human babesiosis has lately increased [60], and this may be related to increasing prevalence of *B. microti* in its reservoir hosts and vectors [61, 62], in which vertical transmission may play a role [9, 10, 18]. Increasing numbers of congenitally acquired human cases of babesiosis warrant further research on the nature of the mechanisms of vertical transmission, and maternal and foetal immunological and hormonal response to infection in order to increase our understanding of these processes and to better inform our strategies for preventing and curing babesiosis more effectively in the future.

## Conclusions

In conclusion, acute *B. microti* infection prevents the initiation of pregnancy and embryonic development during the first trimester, and causes severe complications in foetal BALB/c mice in the second and third trimesters of pregnancy. Chronic *B. microti* infection has no detrimental impact on the initiation and development of pregnancy, but results in congenital infections of the offspring. Further study is required to determine the extent to which maternal anti-babesial immune responses contribute to compromise pregnancy in the murine model of congenital *Babesia* infection.

## Data Availability

All relevant data are included within the article and its additional files.

## Abbreviations

Cl_95_: 95% Confidence interval
DNA: Deoxyribonucleic acid
EDTA: Ethylenediaminetetraacetic acid
GLM: Generalised linear model
iRBC: (Infected) red blood cells
NPI: Non-pregnant, infected
NPU: Non-pregnant, uninfected
NS: not significant
PCR: Polymerase chain reaction
rRNA: Ribosomal ribonucleic acid
SE: Standard error
PU: Pregnant, uninfected

## References

[1] Carlier Y, Truyens C, Deloron P, Peyron F (2012). Congenital parasitic infections: a review. Acta Trop..

[2] Montoya JG, Remington JS (2008). Management of *Toxoplasma gondii* infection during pregnancy. Clin Infect Dis..

[3] Carlier Y, Torrico F (2003). Congenital infection with *Trypanosoma cruzi*: from mechanisms of transmission to strategies for diagnosis and control. Rev Soc Bras Med Trop..

[4] Menendez C, Mayor A (2007). Congenital malaria: the least known consequence of malaria in pregnancy. Semin Fetal Neonatal Med..

[5] Schnieder T, Laabs E-M, Welz C (2011). Larval development of *Toxocara canis* in dogs. Vet Parasitol..

[6] Homer MJ, Aguilar-Delfin I, Telford SR, Krause PJ, Persing DH (2000). Babesiosis. Clin Microbiol Rev..

[7] Tonnetti L, O'Brien SF, Gregoire Y, Proctor MC, Drews SJ, Delage G (2019). Prevalence of *Babesia* in Canadian blood donors: June–October 2018. Transfusion..

[8] Welc-Faleciak R, Bajer A, Bednarska M, Paziewska A, Sinski E (2007). Long term monitoring of *Babesia microti* infection in BALB/c mice using nested PCR. Ann Agric Environ Med..

[9] Tolkacz K, Bednarska M, Alsarraf M, Dwuznik D, Grzybek M, Welc-Faleciak R (2017). Prevalence, genetic identity and vertical transmission of Babesia microti in three naturally infected species of vole, *Microtus* spp. (Cricetidae). Parasit Vectors..

[10] Tufts DM, Diuk-Wasser MA (2020). Vertical transmission: a vector-independent transmission pathway of *Babesia microti* in the natural reservoir host *Peromyscus leucopus*. J Infect Dis..

[11] Bloch EM, Kumar S, Krause PJ. Persistence of *Babesia microti* infection in humans. Pathogens. 2019;8. 10.3390/pathogens8030102 PMC6789900 31319461

[12] De Vos AJ, Imes GD, Cullen JS (1976). Cerebral babesiosis in a new-born calf. Onderstepoort J Vet Res..

[13] Klinger Y, Ben Yossef H. Case of intra-uterine infection with *Babesiella berbera*. [Cattle]. 1972.

[14] Neitz WO (1956). Classification, transmission, and biology of piroplasms of domestic animals. Ann N Y Acad Sci..

[15] Yeruham I, Avidar Y, Aroch I, Hadani A (2003). Intra-uterine infection with *Babesia bovis* in a 2-day-old calf. J Vet Med B..

[16] Adaszek L, Obara-Galek J, Piech T, Winiarczyk M, Kalinowski M, Winiarczyk S (2016). Possible vertical transmission of *Babesia canis canis* from a bitch to her puppies: a case report. Vet Med-Czech.

[17] Mierzejewska EJ, Welc-Falęciak R, Bednarska M, Rodo A, Bajer A (2014). The first evidence for vertical transmission of *Babesia canis* in a litter of Central Asian Shepherd dogs. Ann Agric Environ Med..

[18] Tufts DM, Diuk-Wasser MA (2018). Transplacental transmission of tick-borne *Babesia microti* in its natural host *Peromyscus leucopus*. Parasit Vectors..

[19] Bednarska M, Bajer A, Drozdowska A, Mierzejewska EJ, Tolkacz K, Welc-Faleciak R (2015). Vertical transmission of *Babesia microti* in BALB/c mice: preliminary report. PLoS ONE.

[20] Fukumoto S, Suzuki H, Igarashi I, Xuan X (2005). Fatal experimental transplacental *Babesia gibsoni* infections in dogs. Int J Parasitol..

[21] Aderinboye O, Syed SS (2010). Congenital babesiosis in a four-week-old female infant. Pediatr Infect Dis J.

[22] Esernio-Jenssen D, Scimeca PG, Benach JL, Tenenbaum MJ (1987). Transplacental/perinatal babesiosis. J Pediatr..

[23] Feder HM, Lawlor M, Krause PJ (2003). Babesiosis in pregnancy. N Engl J Med..

[24] Fox LM, Wingerter S, Ahmed A, Arnold A, Chou J, Rhein L (2006). Neonatal babesiosis: case report and review of the literature. Pediatr Infect Dis J..

[25] Hachey K, Lewis D, Rodriguez JL, Richardson MW, Johnston AM (2019). Two cases of congenital babesiosis. Open Forum Infect Dis..

[26] Iyer S, Goodman K (2019). Congenital babesiosis from maternal exposure: a case report. J Emerg Med..

[27] Cornett JK, Malhotra A, Hart D (2012). Vertical transmission of babesiosis from a pregnant, splenectomized mother to her neonate. Infect Dis Clin Pract.

[28] New DL, Quinn JB, Qureshi MZ, Sigler SJ (1997). Vertically transmitted babesiosis. J Pediatr..

[29] Sethi S, Alcid D, Kesarwala H, Tolan RW (2009). Probable congenital babesiosis in infant, New Jersey, USA. Emerg Infect Dis..

[30] Turner CM, Cox FE (1985). The stability of *Babesia microti* infections after prolonged passage, a comparison with a recently isolated strain. Ann Trop Med Parasitol..

[31] McLean AC, Valenzuela N, Fai S, Bennett SA. Performing vaginal lavage, crystal violet staining, and vaginal cytological evaluation for mouse estrous cycle staging identification. J Vis Exp. 2012;e4389. 10.3791/4389 PMC3490233 23007862

[32] Bancroft JD, Layton C, Suvarna SK, Layton C, Bancroft JD (2019). 10—The hematoxylins and eosin. Bancroft's theory and practice of histological techniques (Eighth Edition).

[33] Bonnet S, Jouglin M, L'Hostis M, Chauvin A (2007). *Babesia* sp. EU1 from roe deer and transmission within *Ixodes ricinus*. Emerg Infect Dis..

[34] Rohlf FJ, Sokal RR (1995). Statistical tables.

[35] Vannier E, Krause PJ (2012). Human babesiosis. N Engl J Med..

[36] Luckett R, Rodriguez W, Katz D (2014). Babesiosis in pregnancy. Obstet Gynecol..

[37] Gulersen M, Brost BC, Bobrovnikov V, Bornstein E (2016). Acute babesiosis in pregnancy: a novel imitator of hemolysis, elevated liver enzymes, and low platelet count syndrome. Obstet Gynecol..

[38] Oduola AMJ, Holbrook TW, Galbraith RM, Bank H, Spicer SS (1982). Effects of malaria (*Plasmodium berghei*) on the maternal-fetal relationship in mice. J Protozool..

[39] Poovassery J, Moore JM (2006). Murine malaria infection induces fetal loss associated with accumulation of *Plasmodium chabaudi* AS-infected erythrocytes in the placenta. Infect Immun..

[40] Remington JS, McLeod R, Thulliez P, Desmonts G, Remington JS, Klein JO, Wilson CB, Baker CJ (2006). Toxoplasmosis. Infectious diseases of the fetus and newborn infant.

[41] Torrico F, Alonso-Vega C, Suarez E, Rodrigez P, Torrico M-C, Drmaix M (2004). Maternal *Trypanosoma cruzi* infection. Pregnancy outcome, morbidity, and mortality of congenitally infected and non-infected newborns in Bolivia. Am J Trop Med Hyg..

[42] Menendez C (1995). Malaria during pregnancy: a priority area of malaria research and control. Parasitol Today..

[43] McGready R, Lee SJ, Wiladphaingern J, Ashley EA, Rijken MJ, Boel M (2012). Adverse effects of *falciparum* and *vivax* malaria and the safety of antimalarial treatment in early pregnancy: a population-based study. Lancet Infect Dis..

[44] Nosten F, McGready R, Simpson JA, Thwai KL, Balkan S, Cho T (1999). Effects of *Plasmodium vivax* malaria in pregnancy. Lancet.

[45] Suarez CE, Alzan HF, Silva MG, Rathinasamy V, Poole WA, Cooke BM (2019). Unravelling the cellular and molecular pathogenesis of bovine babesiosis: is the sky the limit?. Int J Parasitol..

[46] Allred DR (2003). Babesiosis: persistence in the face of adversity. Trends Parasitol..

[47] Allred DR, Al-Khedery B (2004). Antigenic variation and cytoadhesion in *Babesia bovis* and *Plasmodium falciparum*: different logics achieve the same goal. Mol Biochem Parasitol..

[48] Cottrell G, Moussiliou A, Luty AJ, Cot M, Fievet N, Massougbodji A (2015). Submicroscopic *Plasmodium falciparum* infections are associated with maternal anemia, premature births, and low birth weight. Clin Infect Dis..

[49] Tagbor H, Bruce J, Browne E, Greenwood B, Chandramohan D (2008). Malaria in pregnancy in an area of stable and intense transmission: is it asymptomatic?. Trop Med Int Health..

[50] Mockenhaupt FP, Bedu-Addo G, Junge C, Hommerich L, Eggelte TA, Bienzle U (2007). Markers of sulfadoxine-pyrimethamine-resistant *Plasmodium falciparum* in placenta and circulation of pregnant women. Antimicrob Agents Chemother..

[51] Mayengue PI, Rieth H, Khattab A, Issifou S, Kremsner PG, Klinkert MQ (2004). Submicroscopic *Plasmodium falciparum* infections and multiplicity of infection in matched peripheral, placental and umbilical cord blood samples from Gabonese women. Trop Med Int Health..

[52] Adamson SL, Lu Y, Whiteley KJ, Holmyard D, Hemberger M, Pfarrer C (2002). Interactions between trophoblast cells and the maternal and fetal circulation in the mouse placenta. Dev Biol..

[53] Joseph JT, Purtill K, Wong SJ, Munoz J, Teal A, Madison-Antenucci S (2012). Vertical transmission of *Babesia microti*, United States. Emerg Infect Dis..

[54] Day MJ (2007). Immune system development in the dog and cat. J Comp Pathol..

[55] Telford SRI, Spielman A, Collier L, Balows A, Sussman M (1988). Babesiosis of humans. Topley & Wilson's microbiology and microbial infection.

[56] Spielman A, Etkind P, Piesman J, Ruebush TK, Juranek DD, Jacobs MS (1981). Reservoir hosts of human babesiosis on Nantucket Island*. Am J Trop Med Hyg..

[57] Wright IG, Goodger BV, Buffington GD, Clark IA, Parrodi F, Waltisbuhl DJ (1989). Immunopathophysiology of babesial infections. Trans R Soc Trop Med Hyg..

[58] Dao AH, Eberhard ML (1996). Pathology of acute fatal babesiosis in hamsters experimentally infected with the WA-1 strain of *Babesia*. Lab Invest..

[59] Schetters TPM, Kleuskens J, Scholtes N, Gorenflot A (1998). Parasite localization and dissemination in the *Babesia*-infected host. Ann Trop Med Parasitol..

[60] Krause PJ (2019). Human babesiosis. Int J Parasitol..

[61] Diuk-Wasser MA, Liu Y, Steeves TK, Folsom-O'Keefe C, Dardick KR, Lepore T (2014). Monitoring human babesiosis emergence through vector surveillance New England, USA. Emerg Infect Dis..

[62] Goethert HK, Molloy P, Berardi V, Weeks K, Telford SR (2018). Zoonotic *Babesia microti* in the northeastern US: evidence for the expansion of a specific parasite lineage. PLoS ONE.

